# Bariatric Surgery Normalizes Protein Glycoxidation and Nitrosative Stress in Morbidly Obese Patients

**DOI:** 10.3390/antiox9111087

**Published:** 2020-11-04

**Authors:** Barbara Choromańska, Piotr Myśliwiec, Magdalena Łuba, Piotr Wojskowicz, Hanna Myśliwiec, Katarzyna Choromańska, Jacek Dadan, Małgorzata Żendzian-Piotrowska, Anna Zalewska, Mateusz Maciejczyk

**Affiliations:** 1 Department of General and Endocrine Surgery, Medical University of Bialystok, 24a M. Sklodowskiej-Curie Street, 15-276 Bialystok, Poland; piotr.a.mysliwiec@gmail.com (P.M.); ananau@wp.pl (M.Ł.); pwojsk@wp.pl (P.W.); jacdad@poczta.onet.pl (J.D.); 2 Department of Dermatology and Venereology, Medical University of Bialystok, 14 Żurawia Street, 15-540 Bialystok, Poland; hanna.mysliwiec@gmail.com; 3 Department of Oral Surgery, Medical University of Gdansk, 7 Dębinki Street, 80-211 Gdansk, Poland; kasia24_89@o2.pl; 4 Department of Hygiene, Epidemiology and Ergonomics, Medical University of Bialystok, 2c Mickiewicza Street, 15-233 Bialystok, Poland; mzpiotrowska@gmail.com; 5 Experimental Dentistry Laboratory, Medical University of Bialystok, 24a M. Sklodowskiej-Curie Street, 15-274 Bialystok, Poland; azalewska426@gmail.com

**Keywords:** morbid obesity, bariatric surgery, protein glycoxidation, nitrosative stress

## Abstract

The results of recent studies indicate the key role of nitrosative stress and protein oxidative damage in the development of morbid obesity. Nevertheless, the effect of bariatric surgery on protein oxidation/glycation and nitrosative/nitrative stress is not yet known. This is the first study evaluating protein glycoxidation and protein nitrosative damage in morbidly obese patients before and after (one, three, six and twelve months) laparoscopic sleeve gastrectomy. The study included 50 women with morbid obesity as well as 50 age- and gender-matched healthy controls. We demonstrated significant increases in serum myeloperoxidase, plasma glycooxidative products (dityrosine, kynurenine, N-formyl-kynurenine, amyloid, Amadori products, glycophore), protein oxidative damage (ischemia modified albumin) and nitrosative/nitrative stress (nitric oxide, peroxy-nitrite, S-nitrosothiols and nitro-tyrosine) in morbidly obese subjects as compared to lean controls, whereas plasma tryptophan and total thiols were statistically decreased. Bariatric surgery generally reduces the abnormalities in the glycoxidation of proteins and nitrosative/nitrative stress. Noteworthily, in the patients with metabolic syndrome (MS+), we showed no differences in most redox biomarkers, as compared to morbidly obese patients without MS (MS−). However, two markers: were able to differentiate MS+ and MS− with high specificity and sensitivity: peroxy-nitrite (>70%) and S-nitrosothiols (>60%). Further studies are required to confirm the diagnostic usefulness of such biomarkers.

## 1. Introduction

Obesity is the leading problem for public health worldwide. This multifactorial disease increases the risk of hypertension, cardiovascular disease, type 2 diabetes (T2DM) and cancer [1,2]. The patho-mechanism of obesity and its metabolic complications has not been fully understood. We still do not know why some morbidly obese patients develop metabolic syndrome (MS), whereas others do not. Adipose tissue distribution is regulated by the interaction of many proteins and hormones including leptin, adiponectin, visfatin, resistin and ghrelin [3,4]. However, recent studies also indicate the critical role of oxidative and nitrosative stress in the pathogenesis of obesity and its related complications [5,6,7,8,9]. Excessive production of reactive oxygen (ROS) and nitrogen (RNS) species lead to oxidative/nitrosative damage to proteins, lipids and DNA. Among all cellular biomolecules, proteins are particularly sensitive to oxidation and glycation. The combination of both processes is often referred to as glycoxidation [10,11]. Glycoxidation of proteins causes their denaturation, fragmentation and aggregation as well as modification/loss of biological function [11,12]. This results in an increase in oxidation/glycation protein products and quenching of the tryptophan fluorescence [10]. Interestingly, the factor with the greatest potential for protein oxidation is peroxy-nitrite [13]. This compound reacts with amino acids (e.g., tyrosine, cysteine and tryptophan), resulting in the formation of carbonyl groups, dimerization, nitration and nitrosylation of proteins. The modification of proteins can lead to inactivation of multiple signaling pathways, initiation of inflammatory processes or apoptosis/cell death [14,15]. Until now, the intensity of protein glycoxidation in morbid obesity has not been determined, especially in patients with concomitant MS. There is also few long-term studies evaluating redox homeostasis in obese people.

The scale of the obesity problem is reflected in the increase of bariatric operations observed in recent years [16]. Currently, bariatric surgery is not only the most effective method of treating obesity, but also its metabolic complications such as hypertension, T2DM and MS [17,18,19]. The exact mechanism of postoperative changes is still unclear. In our previous studies we have shown disturbances in enzymatic and non-enzymatic antioxidant systems as well as increased oxidative damage to cellular lipids and nucleic acids, which partially normalize after bariatric surgery [5,6]. However, the effect of bariatric surgery on oxidation/glycation of proteins is not yet known. Similarly, there are no data on nitrosative stress and protein glycoxidation in obese patients as compared to those with obesity and metabolic syndrome. To our knowledge, this is the first study evaluating myeloperoxidase activity, protein glycoxidation, protein oxidative damage and nitrosative/nitrative stress in morbidly obese patients before as well as one, three, six and twelve months after bariatric surgery. For this purpose, we have chosen the most frequently used redox biomarkers [20,21,22].

## 2. Materials and Methods

### 2.1. Patients

The study consisted of 50 morbidly obese patients (body mass index (BMI) > 40 kg/m^2^), all women (OB) aged from 28 to 56, who underwent elective bariatric surgery at the 1st Department of General and Endocrine Surgery at the University Hospital in Bialystok, Poland. The patients were classified into two subgroups: morbidly obese patients without metabolic syndrome (MS−) (*n =* 25) and morbidly obese patients with metabolic syndrome (MS+) (*n* = 25). MS was diagnosed according to the International Diabetes Federation Guidelines [1]. The blood samples for testing were collected before (OB 0), as well as 1 month (OB 1), 3 months (OB 3), 6 months (OB 6) and 12 months (OB 12) after obesity surgery. The study was conducted in accordance with the Guidelines for Good Clinical Practice and the Declaration of Helsinki, and the protocol was approved by the Ethics Committee of the Medical University of Bialystok (permission code: R-I-002/69/2012 and R-I-002/475/2019). All patients gave their informed consent for inclusion and participation in the study. In MS+ group 23 women had hypertension and 11 had type 2 diabetes mellitus (T2DM). Hypertension was defined according to World Health Organization: blood value of the systolic (SBP) and/or diastolic (DBP) blood pressure 140/90 (mmHg) or above on two different days. Blood pressure was measured on the non-dominant upper arm two times at intervals of several minutes using Diagnosis UA-651 A&D Medical apparatus. The cuff was put on so that it clings tightly to the exposed arm, about 2−3 cm above the elbow flexion. Obese patients with hypertension received the following medication: ACE inhibitors: (perindopril, lisinopril); β blockers (bisoprolol, metoprolol); diuretics (indapamide); calcium channel blockers (amlodipine), whereas patients with T2DM took metformin, gliclazide and/or insulin. None of the patients received β blockers with a nitric oxide donative action (e.g., nebivolol), nitrates nor captopril.

The control group included 50 healthy lean women (BMI < 25 kg/m^2^, aged 28 to 56) treated in q Specialist Dental Clinic at the Medical University of Bialystok, Poland. Only subjects with blood count and biochemical blood parameters (Na^+^, K^+^, INR, ALT, AST and creatinine) within the reference range were enrolled in the study.

Exclusion criteria for both lean and morbidly obese women were as follows: malignancy, acute inflammatory diseases, infectious diseases (HIV/AIDS, hepatitis A, B, or C), autoimmune diseases (Crohn’s and Hashimoto’s disease, ulcerative colitis,), cardiovascular diseases (with the exception of arterial hypertension in the OB group), metabolic diseases, such as osteoporosis, type 1 diabetes, mucopolysaccharidosis and gout), digestive, respiratory or genitourinary systems diseases. All subjects were instructed not to take antibiotics, nonsteroidal anti-inflammatory drugs, glucocorticosteroids and antioxidant supplements (including iron preparations) for three months prior to bariatric surgery. Nevertheless, after laparoscopic sleeve gastrectomy, mineral and vitamin supplementation have been implemented in all patients (WLS Optimum, FitForMe©). All patients declared not smoking nor drinking alcohol for three months prior to blood sampling.

### 2.2. Blood Collection and Laboratory Measurements

Blood samples were collected from patients after overnight fasting into serum and ethylenediaminetetraacetic acid (EDTA)-coated tubes (S-Monovette SARSTEDT) and centrifuged at 4 °C and 4000 rpm for 10 min. For twenty-four hours prior to blood sampling, patients had not performed any intense physical activity. Butylated hydroxytoluene (10 μL 0.5 M BHT/1 mL serum/plasma) was added to all samples to protect against oxidation [23]. The serum and plasma samples were stored at −80°C for further analysis. The Abbott analyzer (Abbott Diagnostics, Wiesbaden, Germany) was used to assay the blood counts and biochemical laboratory parameters. Homeostatic model assessment index (HOMA-IR) was calculated according to Matthews et al. [24].

### 2.3. Redox Assays

All reagents (unless otherwise stated) were purchased from Sigma-Aldrich Nümbrecht, Germany and Sigma-Aldrich Saint Louis, MO, USA. The absorbance and fluorescence were measured using Infinite M200 PRO Multimode Microplate Reader (Tecan Group Ltd., Männedorf, Switzerland). Redox assays were performed in duplicate samples. Due to the high number of determinations, a detailed description of biochemical methods was given in references. The results were standardized to 1 mg of total protein. The total protein concentration was analyzed using a commercial kit (Thermo Scientific PIERCE BCA Protein Assay; Rockford, IL, USA), according to manufacturer’s instructions.

### 2.4. Myeloperoxidase Activity

Serum myeloperoxidase (MPO) activity was measured colorimetrically using sulfanilamide, ortho-dianisidinedihydrochloride, hexadecyltrimethylammonium, and hydrogen peroxide [25]. The absorbance was analyzed at 450 nm.

### 2.5. Protein Glycoxidation

Fluorescence assessment of protein oxidative modifications was performed. For this purpose, dityrosine, kynurenine, N-formyl-kynurenine and tryptophan contents were measured fluorimetrically. Immediately before determination, blood samples were diluted in 0.1 M H_2_SO_4_ (1:5, *v*/*v*) [11]. The characteristic fluorescence at 330/415, 365/480, 325/434 and 295/340 nm, respectively, was measured in 96-well black-bottom microplates [26]. The results were expressed in arbitrary fluorescence units (AFU)/mg protein.

The formation of amyloid cross-β structure was analyzed colorimetrically using thioflavin T [27]. The characteristic fluorescence was measured at 435/485 nm.

The formation of Amadori product was analyzed colorimetrically using nitro blue tetrazolium (NBT) assay [28]. The absorbance was measured at 525 nm and extinction coefficient of 12,640 cm^−1^ mol^−1^ l for monoformazan was used.

The formation of novel glucose-derived fluorescence, termed glycophore, was assessed. Immediately before determination, blood samples were diluted in 0.1 M H_2_SO_4_ (1:5, *v*/*v*) [11]. The characteristic fluorescence of pyraline, pentosidine, furyl-furanyl-imidazole (FFI) and carboxymethyl lysine (CML) was analyzed at 350/440 nm in 96-well black-bottom microplates [29].

The precisions of these measurements, expressed as coefficients of variation (CV), were <4% (dityrosine, kynurenine, N-formyl-kynurenine, tryptophan and Amadori product), <5% (glycophore), and <6% (amyloid-β).

### 2.6. Protein Oxidative Damage

The concentration of total thiols was determined colorimetrically at 420 nm using Ellman’s reagent [30]. The concentration of thiol groups was calculated from the calibration curve using reduced glutathione (GSH) as a standard.

Serum ischemia modified albumin (IMA) concentration was measured colorimetrically. The method is based on the measurement of the exogenous cobalt (Co^2+^) binding facility of the human serum albumin [31]. The absorbance was assessed at 470 nm.

The precision of these measurements, expressed as coefficients of variation (CV), were <4% (total thiols) and <5% (IMA).

### 2.7. Nitrosative/Nitrative Stress

Plasma nitric oxide (NO) concentration was determined indirectly by measuring its stable decomposition products NO_3_^-^ and NO_3_^-^. For this purpose, sulfanilamide and NEDA·2 HCl (N-(1-naphthyl)-ethylenediamine dihydrochloride) were used [32,33]. The absorbance was analyzed at 490 nm.

Plasma peroxy-nitrite concentration was measured colorimetrically based on peroxy-nitrite-mediated nitration resulting in the nitrophenol formation [34]. The absorbance was analyzed at 320 nm.

Plasma S-nitrosothiols concentration was measured colorimetrically based on the reaction of the Griess reagent with Cu^2+^ ions [33,35]. The absorbance was analyzed at 490 nm.

Plasma nitro-tyrosine concentration was analyzed using an ELISA commercial kit (Immundiagnostik AG; Bensheim, Germany), according to the manufacturer’s instructions.

The precisions of these measurements, expressed as coefficients of variation (CV), were <5% (S-nitrosothiols, nitro-tyrosine), <6% (peroxy-nitrite) and <7% (NO).

### 2.8. Statistics

GraphPad Prism 8.3.0 (GraphPad Software, Inc., San Diego, CA, USA) was used for statistical data analysis. The distribution of results was assessed using the Shapiro–Wilk test, while the Leven test was used to assess the homogeneity of variance. Due to lack of normal distribution of results, ANOVA Kruskal–Wallis test and Dunn’s post hoc test were used for comparison of quantitative variables. Multiplicity adjusted p-value was also calculated. Correlations between redox biomarkers and clinical parameters were assessed using Spearman correlation coefficient. The value of redox biomarkers in predicting the risk of MS development was determined using ROC (Receiver Operating Characteristic) analysis analyzing the area under the curve (AUC). The cut-off point with the highest sensitivity and specificity was determined. The test probability p (materiality limit) was established as 0.05 (two-sided).

The number of patients was calculated a priori based on previous preliminary study. The test power was 0.9 and the minimum number of patients in group was 37 (ClinCalc online calculator).

## 3. Results

### 3.1. General Characteristics

Table 1 shows a comparison of the clinical and laboratory characteristics of the lean controls (C) and morbidly obese patients (OB) before and after sleeve gastrectomy. We found markedly higher values in body mass index (BMI) and waist-hip ratio (WHR), as well as in diastolic (DBP) and systolic (SBP) blood pressure, glucose, insulin, homeostatic model assessment of insulin resistance (HOMA-IR), total cholesterol, low-density lipoprotein (LDL), uric acid (UA), urea, C-reactive protein (CRP) and white blood cell count (WBC) in morbidly obese patients as compared with lean controls, whereas high-density lipoprotein (HDL) levels were decreased. After bariatric surgery, all parameters gradually returned to the reference range or improved (BMI, WHR) (Table 1). The clinical characteristics of MS+ and MS− are presented in the Supplementary Material (Table S1).

### 3.2. Myeloperoxidase Activity

**Figure 1:**
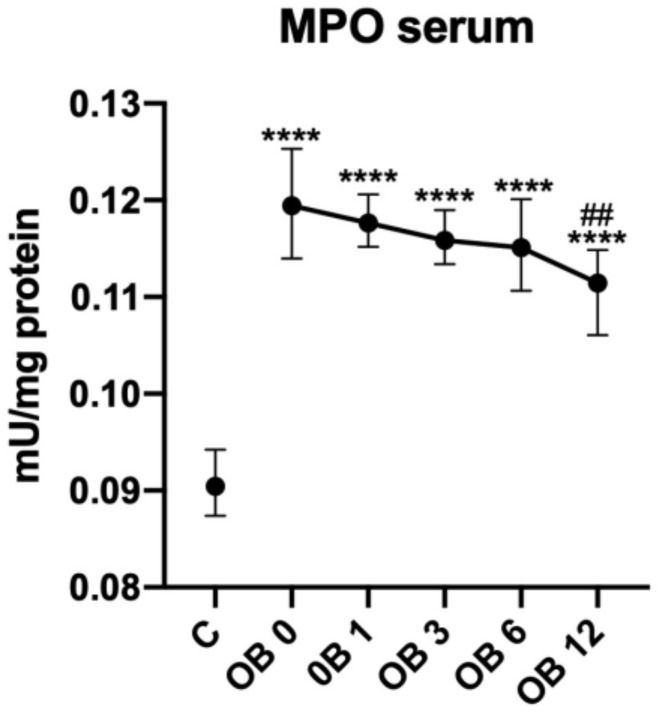
Serum myeloperoxidase (MPO) activity of control (C) and morbid obesity (OB); data given as median and 95%Cl; **** *p* < 0.0001 indicates significant differences from the control (C); ^##^ *p* < 0.01 indicates significant differences from the morbid obesity before laparoscopic sleeve gastrectomy (OB 0); morbidly obese patients before (OB 0), as well as 1 month (OB 1), 3 months (OB 3), 6 months (OB 6) and 12 months (OB 12) after sleeve gastrectomy; myeloperoxidase (MPO).

The serum activity of myeloperoxidase (MPO) was significantly higher in morbidly obese patients prior to (+33%, *p* < 0.0001), as well as after bariatric treatment: OB 1 (+33%, *p* < 0.0001), OB 3 (+33%, *p* < 0.0001), OB 6 (+33%, *p* < 0.0001) and OB 12 (+22%, *p* < 0.0001) in comparison with lean controls. It was only twelve months after surgery when the activity of MPO diminished slightly (−9%, *p* = 0.0095) as compared to preoperative level (Figure 1).

### 3.3. Protein Glycoxidation

**Figure 2:**
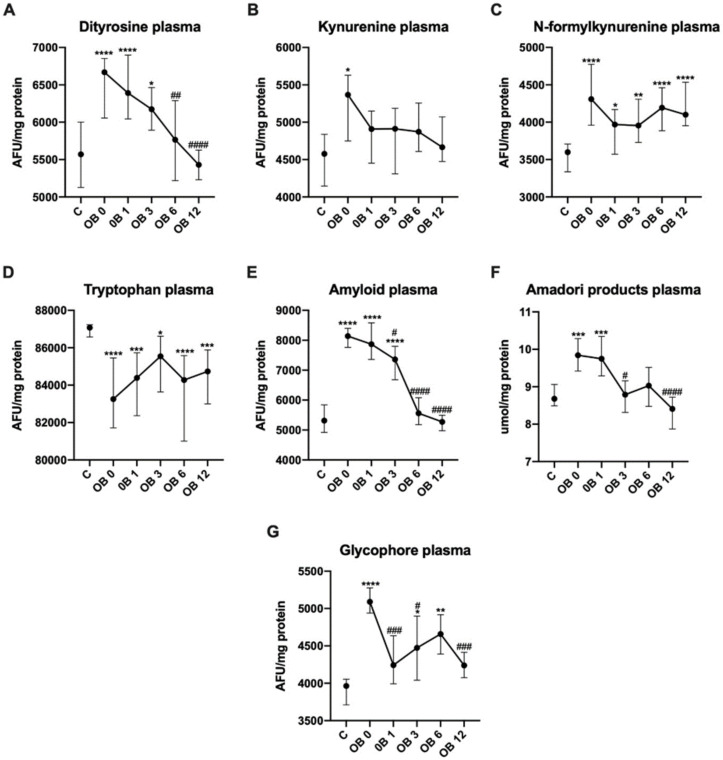
Plasma content of dityrosine (**A**), kynurenine (**B**), N-formyl-kynurenine (**C**), tryptophan (**D**), amyloid (**E**) and glycophore (**G**), as well as plasma concentration of Amadori products (**F**) of control (C) and morbid obesity (OB); data given as median and 95% Cl; * *p* < 0.05, ** *p* < 0.01, *** *p* < 0.001, **** *p* < 0.0001 indicate significant differences from the control (C); # *p* < 0.05, ## *p* < 0.01, ### *p* < 0.001, #### *p* < 0.0001 indicate significant differences from morbid obesity before laparoscopic sleeve gastrectomy (OB 0); morbidly obese patients before (OB 0), as well as 1 month (OB 1), 3 months (OB 3), 6 months (OB 6) and 12 months (OB 12) after sleeve gastrectomy.

The plasma content of dityrosine was significantly increased in OB before (+20%, *p* < 0.0001), as well as 1 (+15%, *p* < 0.0001) and 3 (+11%, *p* = 0.0135) months after bariatric surgery as compared to lean controls. Further on, the content of dityrosine decreased at 6 (−14%, *p* = 0.0096) and 12 (−19%, *p* < 0.0001) months as compared to preoperative values (Figure 2A).

We observed increased kynurenine plasma content in OB (+17%, *p* = 0.0206) before laparoscopic sleeve gastrectomy in comparison with the controls (Figure 2B).

The plasma content of N-formyl-kynurenine was markedly higher in OB 0 (+20%, *p* < 0.0001), OB 1 (+10%, *p* = 0.0341), OB 3 (+10%, *p* = 0.0075), OB 6 (+17%, *p* < 0.0001) and OB 12 (+14%, *p* < 0.0001) subgroups than lean patients (Figure 2C).

We found decreased plasma tryptophan content in morbidly obese women before (−5%, *p* < 0.0001), as well as after bariatric surgery: OB 1 (−4%, *p* = 0.0001), OB 3 (−2%, *p* = 0.019), OB 6 (−4%, *p* < 0.0001) and OB 12 (−3%, *p* = 0.0007) as compared to healthy controls (Figure 2D).

In plasma of morbidly obese patients, the content of amyloid was greater before (+53%, *p* < 0.0001), 1 (+48% *p* < 0.0001) and 3 (+39%, *p* < 0.0001) months after bariatric treatment in comparison with lean patients. We observed decline of amyloid content as compared to OB 0 subgroup at 3 (−10%, *p* = 0.0278), 6 (−32%, *p* < 0.0001) and 12 (−36%, *p* < 0.0001) months after surgery, (Figure 2E).

Morbidly obese patients had higher plasma concentrations of Amadori products than lean controls before (+13%, *p* = 0.007) and 1 month (+11%, *p* = 0.006) after obesity surgery. A decrease was noted at 3 (−11%, *p* = 0.016) and 12 (−15%, *p* < 0.0001) months after surgery in comparison to OB 0 (Figure 2F).

We found greater content of plasma glycophore in OB group before (+28%, *p* < 0.0001), as well as 3 (+13%, *p* + 0.471) and 6 (+18%, *p* = 0.0019) months after bariatric surgery than controls. As compared to OB 0, content was reduced at 1 (−17%, *p* = 0.0003), 3 (−13%, *p* = 0.0215) and 12 (−17%, *p* = 0.0002) months after bariatric treatment (Figure 2G).

### 3.4. Protein Oxidative Damage

**Figure 3:**
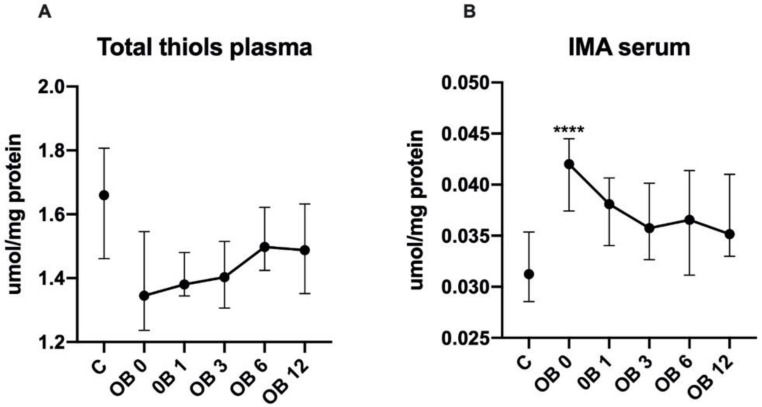
Plasma concentration of total thiols (**A**) and IMA (**B**) of the control (C) and morbid obesity (OB); Data given as median and 95% Cl; *** *p* < 0.001; morbidly obese patients before (OB 0), as well as 1 month (OB 1), 3 months (OB 3), 6 months (OB 6) and 12 months (OB 12) after sleeve gastrectomy; ischemia modified albumin (IMA).

There were no statistically significant differences in the plasma concentrations of total thiols in morbidly obese women as compared to controls (Figure 3A).

The plasma concentrations of ischemia modified albumin (IMA) were greater in morbidly obese patients before (+35%, *p* < 0.0001) sleeve gastrectomy than in healthy controls (Figure 3B).

### 3.5. Nitrosative Stress

**Figure 4:**
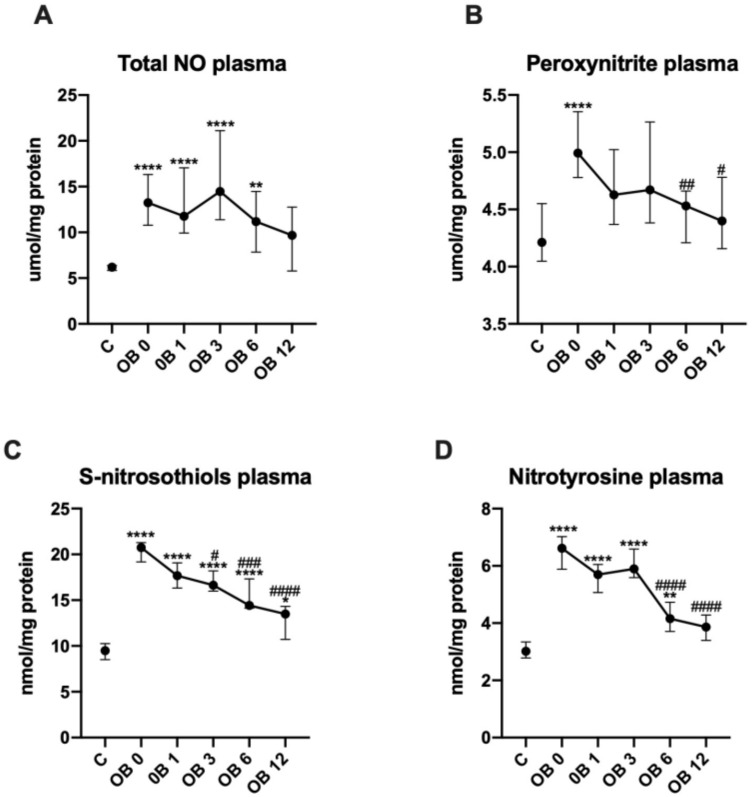
Plasma concentration of total nitric oxide (NO; **A**), peroxy-nitrite (**B**), S-nitrosothiols (**C**) and nitro-tyrosine (**D**) of control (C) and morbid obesity (OB); data given as median and 95% Cl; * *p* < 0.05; ** *p* < 0.01, *** *p* < 0.001, ****p < 0.0001 indicate significant differences from control (C); # *p* < 0.05, ## *p* < 0.01, ### *p* < 0.001, #### *p* < 0.0001 indicate significant differences from morbid obesity before laparoscopic sleeve gastrectomy (OB 0); morbidly obese patients before (OB 0), as well as 1 month (OB 1), 3 months (OB 3), 6 months (OB 6) and 12 months (OB 12) after sleeve gastrectomy; myeloperoxidase (MPO), total nitric oxide (NO).

We observed markedly higher plasma concentration of total NO in morbidly obese patients before (+110%, *p* < 0.0001) bariatric surgery as compared to control group. Moreover, the concentration of total NO remained elevated after laparoscopic sleeve gastrectomy: OB 1 (+94, *p* < 0.0001), OB 3 (+126%, *p* < 0.0001) and OB 6 (+77%, *p* = 0.0012) (Figure 4A).

Figure 4B shows greater plasma concentrations of peroxynitrite in OB 0 (+19%, *p* < 0.0001) than in control group. Starting at half a year after obesity surgery, peroxynitrite levels diminished: OB 6 (−10%, *p* = 0.003) and OB 12 (−12%, *p* = 0.0153) (Figure 4B).

We found greater plasma concentrations of S-nitrosothiols in OB O (+121%, *p* < 0.0001) subgroups than in lean controls. After the operation S-nitrosothiol concentrations gradually decreased: at 3 (−19%, *p* = 0.0154), 6 (−34%, *p* = 0.0001) and 12 months (−38%, *p <* 0.0001), still remaining higher than in control group: OB1 (+89%, *p* < 0.0001), OB 3 (+79%, *p* < 0.0001), OB 6 (+47%, *p* < 0.0001) and OB 12 (+37%, *p* = 0.0339) patients (Figure 4C).

The plasma concentrations of nitrotyrosine were significantly increased in morbidly obese patients before (+120%, *p* < 0.0001), as well as after bariatric surgery: OB 1 (+90%, *p* < 0.0001), OB 3 (+97%, *p* < 0.0001) and OB 6 (+40%, *p* = 0.0025) as compared to lean subjects. As compared to preoperative values, nitrotyrosine concentrations decreased at 6 (−36%, *p* < 0.0001) and 12 (−41%, *p* < 0.0001) months after bariatric treatment (Figure 4D).

### 3.6. Comparison between MS− and MS+

When comparing the results in morbidly obese patients without metabolic syndrome (MS−) and morbidly obese patient with metabolic syndrome (MS+) surprisingly, generally we found no statistically significant differences. We demonstrated some minor changes. There were higher plasma concentrations of Amadori products in MS+ 1 (+6%, *p* = 0.0166) and MS+ 6 (+15%, *p* = 0.0016) than in MS− 1 and MS− 6, respectively. Total NO concentrations were greater in MS+ 6 (+50%, *p* = 0.0483) as compared to MS− 6 subgroups. Plasma content of glycophore was lower in MS+ 3 (−12%, *p* = 0.0494) than MS− 3, and plasma concentrations of total thiols decreased in MS+ 1 (−13%, *p* = 0.0222) in comparison with MS− 1 (Table 2).

The most relevant difference we discovered, pertained to peroxynitrite. Its preoperative concentrations in patients with metabolic syndrome (MS+_0) were 21% greater than in MS− 0 group (*p* < 0.0001). S-nitrosothiols were similarly, although less markedly. elevated (+10%, *p* < 0.001) in MS+ 0 as compared to MS− 0 (Table 2). These differences were no longer visible postoperatively.

### 3.7. ROC Analysis

The above-mentioned differences were confirmed with ROC analysis (Table 3.) Plasma peroxy-nitrite differentiated morbidly obese patients with metabolic syndrome (MS+) from those without metabolic syndrome (MS+) with high sensitivity (72%) and specificity (73%) (AUC 0.8291, *p* = 0.0001) (Table 3).

### 3.8. Correlations

Table S2 depicts correlations between the analyzed nitrosative stress biomarkers and clinical parameters.

The most important associations were found between serum insulin concentrations and plasma peroxy-nitrite (R = 0.326; *p* = 0.031), Amadori products (R = 0.363; *p* = 0.016) and with plasma total thiols (*R* = −0.299; *p* = 0.049). Moreover, there was a positive correlation between the index of insulin resistance (HOMA-IR) and plasma Amadori products (*R* = 0.37; *p* = 0.017), plasma N-formyl-kynurenine (*R* = 0.39; *p* = 0.01) and plasma nitro-tyrosine (*R* = 0.433; *p* = 0.004). Amadori products correlated also positively with BMI (*R* = 0.324; *p* = 0.025) and WHR—a criterium of metabolic syndrome (*R* = 0.356; *p* = 0.013).

We observed a positive association between plasma peroxynitrite, CRP (*R* = 0.311; *p* = 0.035) and UA (*R* = 0.306; *p* = 0.036). Plasma kynurenine negatively correlated with BMI (*R* = −0.33; *p* = 0.022), whereas plasma N-formyl-kynurenine was positive associated with urea (*R* = 0.357; *p* = 0.012).

There was a positive correlation between serum MPO and total cholesterol (*R* = 0.302; *p* = 0.041). Plasma total NO correlated positively with TG (*R* = 0.33; *p* = 0.027) and negatively with HGB (*R* = −0.415; *p* = 0.005). Plasma total thiols were negatively associated with WBC (*R* = −0.354; *p* = 0.013) (Table S2).

Additionally, we checked correlations between the analyzed nitrosative/nitrative stress biomarkers and clinical parameters 12 months after bariatric surgery (Table S3). Plasma kynurenine was positively associated with RBC (*R* = 0.447, *p* = 0.003) and AST (*R* = 0.327, *p* = 0.039), as well as negatively with DBP (*R* = −0.339, *p* = 0.026). Also, plasma N-formyl-kynurenine correlated negatively with DBP (*R* = 0.303, *p* = 0.043) and LDL (*R* = −0.374, *p* = 0.011). We found positive correlations between plasma tryptophan and RBC (*R* = 0.364, *p* = 0.016), plasma Amadori products and BMI (*R* = 0.394, *p* = 0.007), serum MPO and BMI (*R* = 0.386, *p* = 0.011), as well as plasma S-nitrosothiols and UA (*R* = 0.303, *p* = 0.043). The content of dityrosine correlates negatively with weight loss at the end of the experiment (*R* = −0.334, *p* = 0.025) (Table S3).

## 4. Discussion

This is the first study evaluating protein glycoxidation, protein oxidative damage and nitrosative/nitrative stress in morbidly obese patients before and after (one, three, six and twelve months) laparoscopic sleeve gastrectomy. We demonstrated significant increases in plasma glyco-oxidative products, protein oxidation and nitrosative/nitrative stress in morbidly obese subjects as compared to lean controls. Noteworthily, in patients with metabolic syndrome (MS+), we showed no differences in most redox biomarkers, as compared to morbidly obese patients without MS (MS−). However, two markers were able to differentiate MS+ and MS− with high specificity and sensitivity: peroxy-nitrite (>70%) and S-nitrosothiols (>60%). After bariatric surgery physiological redox homeostasis was markedly improved.

The main aim of obesity surgery is not to lose weight to achieve an aesthetic effect, but to improve the metabolic state of obese patients, prolong their live and improve its quality. Bariatric treatment maintains long-term weight loss and reduces the risk of several diseases such as hypertension, T2DM or cardiovascular disorders [18,36]. In this study, we showed that bariatric surgery resulted in the reduction of class 3 obesity to class 1, 12 months after procedure. Along with weight loss in morbidly obese patients, we also observed an improvement in carbohydrate and lipid metabolism.

The results of recent studies indicate the key role of nitrosative/nitrative stress in the development of morbid obesity. It has been shown that the progression of metabolic disturbances accompanying obesity is paralleled by increase in myeloperoxidase activity, NO formation as well as protein nitrosative damage [37]. Importantly, nitrosative stress is linked to endothelial dysfunction and thus excessive platelet aggregation or induction of atherogenesis [38,39]. However, it is still unclear whether bariatric surgery reduces/eliminates nitrosative stress to the level observed in lean control. Due to the short half-life, direct analysis of RNS quantities is virtually impossible. Thus, the evaluation of oxidative/nitrosative stress is more often based on compounds formed by the reaction of ROS/RNS with cellular components. Markers of protein oxidative damage are among the best because proteins are present in every cell. The products of their oxidation are relatively stable and easy to analyze [40].

We have shown that glycoxidation rate (↑dityrosine, ↑kynurenine, ↑N-formyl-kynurenine, ↑amyloid, ↑Amadori products and ↑glycophore) as well as protein oxidative damage (↑IMA) were significantly higher in the preoperative plasma of obese patients as compared to the control group. This fact is not surprising, because in morbidly obese patients many metabolic pathways such as sorbitol, polyol, hexosamine and protein kinase C are activated [41,42]. Under these conditions, glucose autoxidation also occurs, resulting in the formation of reactive ketoaldehyde, which further enhances non-enzymatic glycation [10,43]. Glycoxidation products (especially AGE, advanced glycation end products) can also activate the pro-inflammatory pathway NF-KB, which increases the expression of pro-inflammatory cytokines and chemokines, growth factors, adhesion molecules, and nitric oxide synthases (mainly iNOS) [15,44]. In our study, glycation and oxidation of proteins generally normalize after bariatric surgery; nevertheless, the fluorescence of N-formyl-kynurenine is significantly increased, while the tryptophan content is statistically reduced even one year after surgery. This condition undoubtedly results from the improvement of metabolic parameters after the bariatric treatment. Indeed, in patients after surgery, a better lipid and carbohydrate profile is observed. Nevertheless, the decrease in blood glucose level deserves special attention. Although protein glycation occurs very slowly in vivo, it intensifies dramatically under the hyperglycemic conditions [10,45]. Interestingly, the content of dityrosine correlates negatively with weight loss at the end of the experiment.

Until twelve months after laparoscopic sleeve gastrectomy we also observed a diminished MPO activity; however, this decline is not at the same level of the control group. A slight decrease in MPO expression may be due to the fact that, despite enormous weight loss, patients from the study group were still obese. It has been demonstrated that MPO takes part in the peroxidation of LDL cholesterol, which is embedded in atherosclerotic plaques of blood vessels [46]. MPO also initiates/promotes acute and chronic inflammation, as well as atherosclerosis by the production of pro-oxidants such as HOCl and tyrosyl radicals [47].

In the OB group we also found enhanced NO level which dynamically reduced after the bariatric treatment. Interestingly, total NO concentration was also significantly higher in MS 6 + subgroup than MS 6 -. Although the bioavailability of NO has been shown to be reduced both in the animal model of obesity and diabetes [48], as well as in obese and T2DM patients [49,50], it is worth noting that obesity is an inflammatory disease. Indeed, NO generated by iNOS (inducible nitric oxide synthase) plays a crucial role in inflammatory processes and has the highest capacity to produce NO [51]. Therefore, in morbidly obese patients, chronic inflammation may lead to the increased formation of nitric oxide. NO is also a substrate for peroxy-nitrite formation. ONOO^−^ is a strong oxidant with harmful effect on the cellular biomolecules (e.g., amino acids, lipids and nucleic acids) [52]. Nevertheless, the main target of its action are proteins. Indeed, ONOO^−^ oxidizes thiol groups and tyrosine in signaling proteins/enzymes as well as in proteins involved in energy cell metabolism [53]. In our study, we found greater concentration of ONOO^−^ and nitro-tyrosine in morbidly obese patients before laparoscopic gastrectomy as compared to lean controls. Nevertheless, their levels are gradually reduced after bariatric treatment. Before surgery, we also observed an increase in plasma S-nitrosothiols concentration. This may be an adaptive reaction to the overproduction of RNS in obese patients. Indeed, in biological systems, S-nitrosothiols are involved in transport and cellular storage of nitric oxide [54]. S-nitrosothiols also protect cells against the toxic effects of NO_2_, NO^+^, NO^-^ and ONOO^-^ [54]. Therefore, it is not surprising, that along with the normalization of protein nitrosative/nitrative damage, the concentration of S-nitrosothiols returned to the values found in the lean controls.

Obesity is a disease with complex etiology in which redox imbalance seems to play a special role [55]. Although we are unable to determine whether oxidative stress is the cause or effect of metabolic complications in obesity, this process can exacerbate inflammation as well as disturb the lipid and carbohydrate metabolism. However, we did not show any differences between patients with obesity and metabolic syndrome from patients with obesity alone. The lack of change may result from a small study group, but also from the complexity of metabolic disturbances in obese patients. Additionally, it should be remembered that in our study we measured only circulating redox biomarkers. The evaluation of plasma oxidation/glycation of proteins and nitrosative/nitrative stress may not necessarily reflect metabolic changes in target organs such as adipose tissue, liver or skeletal muscles [56]. However, before the surgery, peroxy-nitrite content was significantly higher in the OB + MS subgroup compared to OB. Similar changes have been observed for S-nitrosothiols. Using the ROC analysis, we have demonstrated the diagnostic usefulness of these parameters in differentiating metabolic disturbances in the course of obesity. Indeed, plasma ONOO^−^ and S-nitrosothiols with high sensitivity and specificity distinguish obese patients from those with OB and MS. The peroxy-nitrite content was also positively correlated with insulin, uric acid and CRP levels. Although the correlations observed are relatively poor, they may suggest the contribution of peroxy-nitrite and S-nitrosothiols in the progression of metabolic changes in obesity. Nowadays, redox biomarkers are increasingly used in differential diagnosis/prediction of the severity of many diseases such as hypertension [9], diabetes [57], chronic heart disease [7], chronic kidney disease [58] and stroke [59]. Nevertheless, in order to confirm the diagnostic usefulness of redox biomarkers in morbid obesity patients, long term observations on a larger number of cases are necessary.

In our work, we did not compare the effect of different techniques of bariatric surgery on the oxidative/nitrosative stress of obese patients. Laparoscopic sleeve gastrectomy (LSG) reduces gastric capacity, which leads to lower intake and absorption of food and decreased secretion of ghrelin. Although laparoscopic sleeve gastrectomy and laparoscopic adjustable gastric banding (RYGBP) are the most standard surgical procedures, biliopancreatic diversion (BPD) and mini gastric bypass (mini-GBP) are among the most effective but also have more side effects [60,61]. In future studies, due to the different mechanism of weight loss, it is necessary to compare various bariatric procedures on cellular redox homeostasis of obese patients.

Despite the undoubted advantages, our study also has some limitations. We assessed only selected biomarkers of glycoxidation and nitrosative/nitrative stress, so we cannot fully characterize the effect of bariatric surgery on redox homeostasis in morbidly obese patients. Furthermore, we are not able to determine the source of NO formation and we have not evaluated the expression of proinflammatory cytokines, which are inseparable from the oxidation and glycation of proteins [62]. Finally, we are not able to eliminate the influence of drugs and diet on the assessed redox biomarkers.

In conclusion, we have shown that protein glycoxidation and nitrosative/nitrative stress is markedly deranged in obese patients. Although oxidative/nitrosative stress contributes to the progression of metabolic changes in obesity [63,64], oxidation and glycoxidation of plasma proteins did not differ between MS+ and MS. Within the broad spectrum of studied parameters, only peroxy-nitrite and S-nitrosothiols differentiated morbidly obese patients with metabolic syndrome from those without. Bariatric surgery generally reduces the abnormalities in the oxidation and glycation of proteins as well as nitrosative/nitrative stress. Further studies are required to confirm the diagnostic usefulness of such biomarkers.

## Figures and Tables

**Table 1:** Clinical characteristics of the control (C) and morbid obesity (OB). Data given as median (lower and upper confidence limit), * *p* < 0.05, ** *p* < 0.01, *** *p* < 0.001, **** *p* < 0.0001 indicate significant differences from the control, # *p* < 0.05, ## *p* < 0.01, ### *p* < 0.001, #### *p* < 0.0001 indicate significant differences from morbid obesity before laparoscopic sleeve gastrectomy (OB 0); before (OB 0); as well as, 1 month (OB 1), 3 months (OB 3), 6 months (OB 6) and 12 months (OB 12) after laparoscopic sleeve gastrectomy; alanine transaminase (ALT), aspartate transaminase (AST), body mass index (BMI), C-reactive protein (CRP), creatinine (Crea), diastolic blood pressure (DBP), high-density lipoprotein (HDL), hemoglobin (HGB), homeostatic model assessment of insulin resistance (HOMA-IR), low-density lipoprotein (LDL), red blood cell count (RBC), systolic blood pressure (SBP), triacylglycerol (TG), uric acid (UA), white blood cell count (WBC), waist-hip ratio (WHR).

	C	OB 0	OB 1	OB 3	OB 6	OB 12	ANOVA
**Age**	45	45					>0.9999
	(41–49)	(41–49)					
**Weight (kg)**	62	124	113	100.5	90 ****^####^	81.5 ***^####^	<0.0001
	(60.32–63)	(121.5–131.1)	(106.4–115.8)	(96.6–104.9)	(86.4–93.2)	(78–84.8)	
**BMI (kg/m^2^)**	23	46 ****	41 ****	37 ****^###^	34 ****^####^	30 ***^####^	<0.0001
	(23–23)	(45–48)	(40–43)	(36–40)	(32–35)	(29–32)	
**Weight loss (kg)**			13	24	33	41.5	<0.0001
			(10.39–13.09)	(21.52–25.3)	(30–34.34)	(38.72–44.03)	
**WHR**	0.72	0.97 ****	0.98 ****	0.97 ****	0.96 ****	0.92 ****^###^	<0.0001
	(0.71–0.72)	(0.96–0.99)	(0.96–0.99)	(0.94–0.99)	(0.93–0.97)	(0.91–0.94)	
**SBP (mmHg)**	120	130 ****	130 ****	130 ****	128 ***	125 **	<0.0001
	(110–120)	(125–140)	(125–140)	(125–135)	(120–135)	(120–130)	
**DBP (mmHg)**	80	85 ****	85 ***	80 *	80 *	80	<0.0001
	(70–80)	(80–90)	(80–90)	(80–90)	(80–85)	(80–85)	
**Glucose (mg/dL)**	76	101 ****	98 ****	93 ****^#^	93 ****	87 ****^####^	<0.0001
	(73–78)	(95–106)	(91–99)	(90–97)	(87–96)	(85–92)	
**Insulin (** **μIU/mL)**	7.6	19 ****	13 ***^####^	9 ^####^	8.6 ^####^	7.8 ^####^	<0.0001
	(7.4–7.8)	(17–22)	(9.8–15)	(7.3–11)	(7.5–9.2)	(6.9–8.5)	
**HOMA–IR**	1.4	4.4 ****	3 ****^##^	2 ***^####^	1.9 ^*####^	1.7 ^####^	<0.0001
	(1.3–1.5)	(4–5.4)	(2.4–3.5)	(1.7–2.4)	(1.7–2.2)	(1.5–1.9)	
**Cholesterol (mg/dL)**	175	198 ****	185 ^#^	177 ^##^	184	175 ^####^	<0.0001
	(170–178)	(186–209)	(175–192)	(173–184)	(174–191)	(167–180)	
**HDL (mg/dL)**	60	46 ****	45 ****	47 ****	50 ***	55 ^#^	<0.0001
	(59–62)	(42–54)	(39–49)	(43–49)	(48–54)	(51–57)	
**TG (mg/dL)**	134	135	126	115	116 **^##^	98 ****^####^	<0.0001
	(130–135)	(125–151)	(107–139)	(103–135)	(99–124)	(85–106)	
**LDL (mg/dL)**	118	137 *	115 ^####^	112 ^####^	109 ^####^	103 **^####^	<0.0001
	(116–120)	(128–148)	(110–120)	(104–119)	(99–118)	(99–113)	
**ALT (IU/L)**	25	27	28	22	20 ^###^	18 ^####^	<0.0001
	(22–27)	(24–30)	(21–35)	(19–24)	(17–21)	(17–20)	
**AST (IU/L)**	23	20	27	19	18 **	18	<0.0001
	(22–26)	(18–24)	(22–32)	(17–21)	(16–20)	(16–25)	
**UA (mg/dL)**	3.9	6.4 ****	5.9 ****	5.1 ****^##^	4.8 *^####^	4.4 ^####^	<0.0001
	(3.8–4.3)	(5.7–7.2)	(5.4–6.2)	(4.8–5.5)	(4.5–5)	(4.1–4.7)	
**Urea (mg/dL)**	25	29 *	26	23 ^##^	25	25 ^#^	0.0053
	(22–27)	(25–32)	(22–28)	(22–26)	(22–27)	(22–27)	
**Crea (mg/dL)**	0.74	0.73	0.76	0.72	0.72	0.75	0.2102
	(0.71–0.76)	(0.71–0.75)	(0.72–0.8)	(0.7–0.76)	(0.69–0.75)	(0.7–0.79)	
**CRP (mg/L)**	5.5	10 *	6	6.7	5.3 ^###^	5.1 ^####^	<0.0001
	(5.3–5.7)	(6.7–12)	(5.1–7.8)	(6–7.1)	(5–6.3)	(4.9–5.3)	
**WBC (10^3^/μL)**	7.5	8.8 **	6.8 ^###^	6.1 ^####^	6.7 ^####^	6.1 ^####^	<0.0001
	(6.8–7.8)	(8.1–9.8)	(6.5–7.6)	(5.8–6.9)	(5.9–7.9)	(5.5–7)	
**RBC (10^6^/μL)**	4.6	4.6	4.7	4.7	4.7	4.6	0.3807
	(4.5–4.7)	(4.4–4.9)	(4.6–4.9)	(4.5–4.8)	(4.4–4.8)	(4.5–4.8)	
**HGB (g/dL)**	14	13	14	13	13	14	0.0355
	(14–14)	(13–14)	(13–14)	(13–14)	(13–14)	(13–14)	
**PLT (10^3^/μL)**	289	258	237 ****	255 **	261	221 ****^#^	<0.0001
	(278–298)	(231–299)	(213–254)	(225–277)	(238–307)	(197–230)	

**Table 2:** Comparison between morbidly obese patients without metabolic syndrome (MS−) and morbidly obese patients with metabolic syndrome (MS+); before (MS− 0, MS+ 0), as well as 1 month (MS− 1, MS+ 1), 3 months (MS− 3, MS+ 3), 6 months (MS− 6, MS+ 6) and 12 months (MS− 12, MS+ 12) after laparoscopic sleeve gastrectomy; data given as median (lower and upper confidence limit); *** *p* < 0.001, ****p < 0.0001 indicate significant differences from the MS− 0, # *p* < 0.05 indicate significant differences from the MS− 1, *p* < 0.05 indicate significant differences from the OB- 3, ~ *p* < 0.05, ~~ *p* < 0.01 indicate significant differences from the MS− 6; ischemia modified albumin (IMA); myeloperoxidase (MPO); nitric oxide (NO).

	MS− 0	MS− 1	MS− 3	MS− 6	MS− 12	MS+ 0	MS+ 1	MS+ 3	MS+ 6	MS+ 12
	*n* = 25					*n* = 25				
**Dityrosine**	5433	6267	6144	5592	5385	5631	6624	6174	5871	5587
**(AFU/mg Protein)**	(4879−6002)	(5820-7069)	(5489-6487)	(5059-6570)	(4912–5647)	(5160–6172)	(5863–7042)	(5901–6578)	(5087–6166)	(4943–5851)
**Kynurenine**	5525	4927	5104	4996	4665	5032	4813	4402	4802	4619
**(AFU/mg protein)**	(4881−5743)	(4238−5231)	(4514−5614)	(4156−5841)	(4501−5842)	(4157–5628)	(3866–5329)	(3916–5173)	(3114–5240)	(3378–5136)
**N–formyl-kynurenine**	4257	3903	3864	4130	4147	4343	4072	4136	4258	4061
**(AFU/mg protein)**	(3858−4774)	(3472−4169)	(3429−4352)	(3775−4454)	(3757−4577)	(3929–4982)	(3431–4533)	(3780–4402)	(3704–4721)	(3932–5162)
**Tryptophan**	83957	82467	86026	85097	84737	82126	85232	84260	83275	84671
**(AFU/mg protein)**	(81854–86712)	(80908–85730)	(84293–87229)	(79487–89554)	(82142–87014)	(78363–85510)	(81778–87357)	(82824–86703)	(80369–85412)	(82712–86292)
**Amyloid**	8220	7359	7404	5564	5135	8095	8570	7210	5557	5303
**(AFU/mg protein)**	(7594–8760)	(6627–8162)	(6284–7800)	(5262–6083)	(4859–5495)	(7498–8528)	(7563–8712)	(6620–8025)	(5034–6389)	(4977–5820)
**Amadori products**	9.5	9.4	8.5	8.5	8	10	10 ^#^	8.9	9.8 ^~~^	8.6
**(µmol/mg protein)**	(8.6–0)	(8.7–9.7)	(8.1–9.1)	(8.2–8.9)	(7–8.9)	(9.5–11)	(9.7–11)	(8.4–10)	(9.2–11)	(8.1–9.1)
**Glycophore**	5237	4137	4799	4688	4233	5039	4339	4228 ^^^	4451	4245
**(AFU/mg protein)**	(4512–5333)	(3919–4601)	(3852–5329)	(4478–4974)	(3805–4551)	(4939–5282)	(3792–4954)	(3848–4637)	(3987–4948)	(4058–4525)
**Total thiols**	1.4	1.5	1.4	1.5	1.5	1.3	1.3 ^#^	1.4	1.5	1.5
**(µmol/mg protein)**	(1.2–1.7)	(1.4–1.6)	(1.3–1.7)	(1.4–1.6)	(1.4–1.7)	(1.2–1.5)	(1.2–1.4)	(1.3–1.6)	(1.4–1.8)	(1.2–1.7)
**IMA**	0.038	0.038	0.034	0.037	0.042	0.043	0.038	0.036	0.034	0.034
**(µmol/mg protein)**	(0.035–0.044)	(0.032–0.044)	(0.031–0.041)	(0.032–0.042)	(0.033–0.051)	(0.039–0.045)	(0.034–0.041)	(0.032–0.052)	(0.03–0.042)	(0.03–0.037)
**MPO**	0.12	0.12	0.12	0.11	0.11	0.12	0.12	0.12	0.12	0.11
**(mU/mg protein)**	(0.11–0.13)	(0.11–0.12)	(0.11–0.12)	(0.11–0.12)	(0.1–0.12)	(0.11–0.13)	(0.11–0.12)	(0.11–0.12)	(0.11–0.13)	(0.1–0.12)
**Total NO**	12	11	12	9.3	6.4	16	15	16	14 ^~^	12
**(µmol/mg protein)**	(11–20)	(8.6–17)	(5.8–23)	(4.5–13)	(3.7–13)	(9.8–27)	(9.5–21)	(8.2–22)	(7.9–20)	(5.8–17)
**Peroxy-nitrite**	4.8	4.6	4.8	4.4	4.5	5.8 ^****^	4.6	4.6	4.6	4.3
**(µmol/mg protein)**	(4.4–5.1)	(4.4–5.2)	(4.1–5.7)	(4.1–4.8)	(4.1–5.2)	(5–6.4)	(4.2–5.3)	(4.4–5.7)	(4.2–4.8)	(4.1–4.8)
**S–nitroso-thiols**	20	18	16	14	13	22 ^***^	18	17	17	14
**(nmol/mg protein)**	(16–21)	(16–21)	(14–18)	(14–16)	(7.9–16)	(20–23)	(16–20)	(16–19)	(13–18)	(9.7–15)
**Nitro-tyrosine**	6.1	5.7	5.9	4.1	4	6.7	5.6	6.2	4.2	3.7
**(nmol/mg protein)**	(5.3–7.2)	(5.2–6.7)	(4.9–6.7)	(3.5–4.8)	(3.1–4.3)	(5.9–7.1)	(4.7–6.4)	(5.7–6.9)	(3.6–4.8)	(3.1–4.3)

**Table 3:** Area under the curve (AUC) of protein glycoxidation and nitrosative stress biomarkers between morbidly obese patients without metabolic syndrome (MS–) and morbidly obese patients with metabolic syndrome (MS+); ischemia modified albumin (IMA); myeloperoxidase (MPO); nitric oxide (NO).

	AUC	95% CI	*P* Value	Cut off	Sensitivity%	95% CI	Specificity%	95% CI
**Dityrosine**	0.54	0.38 to 0.70	0.6208	>5571	60	41% to 77%	60	41% to 77%
**(AFU/mg protein)**								
**Kynurenine**	0.65	0.49 to 0.80	0.0833	<5270	58	39% to 76%	62	43% to 79%
**(AFU/mg protein)**								
**N-formyl-kynurenine**	0.55	0.39 to 0.72	0.5222	>4287	56	37% to 73%	54	35% to 72%
**(AFU/mg protein)**								
**Tryptophan**	0.6	0.44 to 0.76	0.2225	<83402	60	41% to 77%	58	39% to 76%
**(AFU/mg protein)**								
**Amyloid**	0.57	0.40 to 0.73	0.4187	<8155	56	37% to 74%	54	35% to 72%
**(AFU/mg protein)**								
**Amadori products**	0.64	0.48 to 0.80	0.099	>9.921	58	39% to 76%	62	43% to 79%
**(µmol/mg protein)**								
**Glycophore**	0.52	0.35 to 0.68	0.8538	>5061	48	30% to 66%	44	27% to 63%
**(AFU/mg protein)**								
**Total thiols**	0.59	0.43 to 0.76	0.2745	<1.345	58	39% to 76%	58	39% to 76%
**(µmol/mg protein)**								
**IMA**	0.59	0.43 to 0.75	0.2836	>0.0415	62	43% to 79%	58	39% to 76%
**(µmol/mg protein)**								
**MPO**	0.5	0.34 to 0.67	0.9507	<0.1194	50	31% to 69%	50	31% to 69%
**(mU/mg protein)**								
**Total NO**	0.56	0.40 to 0.73	0.4354	>13.28	54	35% to 72%	56	37% to 73%
**(µmol/mg protein)**								
**Peroxy-nitrite**	0.83	0.71 to 0.95	0.0001	>4.984	72	52% to 86%	73	52% to 87%
**(µmol/mg protein)**								
**S-nitroso-thiols**	0.78	0.65 to 0.91	0.0011	>20.51	64	45% to 80%	64	43% to 80%
**(nmol/mg protein)**								
**Nitro-tyrosine**	0.52	0.35 to 0.69	0.8084	>6.400	60	41% to 77%	56	37% to 73%
**(nmol/mg protein)**								

## Supplementary Materials

The following are available online at https://www.mdpi.com/2076-3921/9/11/1087/s1. Table S1: Clinical characteristics of the control (C) morbid obesity without metabolic syndrome (MS−) and morbid obesity with metabolic syndrome (MS+); Table S2: Correlations between the analyzed oxidative/nitrosative stress and clinical parameters in patients with morbid obesity (OB) at the beginning of the study; Table S3: Correlations between the analyzed oxidative/nitrosative stress and clinical parameters in patients with morbid obesity (OB) at the end of the study.
