# Supplementary material for: Bariatric Surgery Normalizes Protein Glycoxidation and Nitrosative Stress in Morbidly Obese Patients
Source: Antioxidants (Basel). 2020 Nov 4;9(11):1087. doi: 10.3390/antiox9111087 (PMC7694407; doi:10.3390/antiox9111087)
Supplement: Supplementary file 1 [file antioxidants-09-01087-s001.pdf]

Table S1. Clinical characteristics of the control (C) morbid obesity without metabolic syndrome (MS-) and morbid obesity with metabolic syndrome (MS+). Data given as median (lower and upper confidence limit),), * p<0.05, ** p<0.01, *** p<0.001, **** p<0.0001 indicate significant differences from the control, # p<0.05, ## p<0.01, ### p<0.001, #### p<0.0001 indicate significant differences from the morbid obesity without metabolic syndrome (MS- 0) patients before bariatric surgery; ^ p<0.05, ^^ p<.0.01 ^^^ p<.0.001 ^^^^ p<.0.0001 indicate significant differences from the morbid obesity with metabolic syndrome (MS+ 0) patients before bariatric surgery, morbidly obese patients without metabolic syndrome (MS-) and morbidly obese patients with metabolic syndrome (MS+), before (MS- 0; MS+ 0) as well as one month (MS- 1; MS+ 1), three months (MS- 3; MS+ 3), six months (MS- 6; MS+ 6)and twelve months (MS- 12; MS+ 12) after laparoscopic sleeve gastrectomy; alanine transaminase (ALT), aspartate transaminase (AST), body mass index (BMI), C-reactive protein (CRP), creatinine (Crea), diastolic blood pressure (DBP), high-density lipoprotein (HDL), hemoglobin (HGB), homeostatic model assessment of insulin resistance (HOMA-IR), low-density lipoprotein (LDL), red blood cell count (RBC), systolic blood pressure (SBP), triacylglycerol (TG), uric acid (UA), white blood cell count (WBC), waist-hip ratio (WHR).

	C	MS- 0	MS+ 0	MS- 1	MS+ 1	MS- 3	MS+ 3	MS- 6	MS+ 6	MS- 12	MS+ 12	P value
Age	45<br>(41.45-45.87)	39<br>(37.8-44.2)	47<br>(43.46-49.18)									0.0854
Weight (kg)	62<br>(60.32-63)	120****<br>(119.7-134.4)	124****<br>(119-131.9)	112****<br>(103.6-115.4)	113****<br>(105-120.3)	98.5****#<br>(93.4-104.9)	102****<br>(95.86-108.8)	89***####<br>(83.92-94)	92****^^^<br>(85.64-95.57)	77####<br>(74.52-83.57)	84.5*^^^^<br>(78.35-88.81)	<0.0001
BMI (kg/m^2)	22.86<br>(21.74-40.17)	44.92****<br>(43.45-47.26)	46.38****<br>(45.49-49.76)	40.39****<br>(38.63-42.78)	41.51****<br>(40.11-45.1)	36.63****<br>(34.58-38.73)	39.67****<br>(36.65-40.94)	32.85**####<br>(31.41-34.88)	34.63**^^^<br>(32.63-36.11)	28.99####<br>(27.88-31.06)	31.23^^^^<br>(29.97-33.56)	<0.0001
Weight loss (kg)				13<br>(9.415-14.01)	12.5<br>(10.1-13.44)	24<br>(20.76-25.93)	24<br>(20.51-26.45)	33.5<br>(29.47-35.61)	32<br>(28.49-35.09)	43<br>(38.49-46.47)	41<br>(36.6-44.12)	<0.0001
WHR	0.716<br>(0.674-0.727)	0.96****<br>(0.934-0.979)	0.978****<br>(0.956-1.004)	0.96****<br>(0.912-0.976)	0.993****<br>(0.970-1.02)	0.944****<br>(0.906-0.969)	0.984****<br>(0.945-0.999)	0.935****<br>(0.909-0.949)	0.963****<br>(0.939-0.986)	0.913**<br>(0.89-0.93)	0.933***<br>(0.911-0.94)	<0.0001
SBP (mmHg)	120<br>(117.7-125.3)	120<br>(121.9-127.3)	140****<br>(134.5-142.3)	120<br>(121.4-127.8)	140****<br>(132.3-139.9)	120<br>(121.5-126.9)	140****<br>(131.5-137.6)	120<br>(120.6-125.6)	135****<br>(129.2-136.2)	120<br>(119.7-123.8)	130****<br>(129.3-135.1)	<0.0001
DBP (mmHg)	80<br>(78.25-89.94)	80<br>(81.45-85.35)	90****<br>(86.98-93.82)	80<br>(78.85-83.76)	90****<br>(86.14-90.38)	80<br>(79.45-83.88)	87.5*<br>(83.35-87.9)	80<br>(79.46-83.04)	87.5*<br>(83.71-88.37)	80<br>(79.5-82.68)	90*<br>(83.46-87.84)	<0.0001
Glucose (mg/dL)	76<br>(74.45-77.93)	96.5****<br>(91.44-101)	106****<br>(101.8-121)	91****<br>(86.46-97.09)	99****<br>(97.14-111.5)	91**<br>(82.07-95.15)	97****<br>(92.78-99.83)	87**<br>(84.16-93.27)	95.5****<br>(90.81-104.9)	84.5<br>(82.62-90.29)	90****^^<br>(87.71-92.69)	<0.0001
Insulin (µIU/mL)	7.6<br>(7.539-7.882)	17.1****<br>(15.53-20.69)	22.5****<br>(19.71-25.2)	10.4##<br>(8.629-12.63)	15.1****<br>(12.29-17.83)	6.9####<br>(6.266-8.786)	10.9*^<br>(9.182-15.83)	8####<br>(6.687-9.15)	8.9^^^^<br>(8.165-10.63)	6.8####<br>(5.61-8.132)	8.4^^^^<br>(7.352-8.6)	<0.0001
HOMA-IR	1.44<br>(1.401-1.501)	4.089****<br>(3.627-5.109)	5.903****<br>(4.961-6.33)	2.371##<br>(1.829-2.942)	3.678****<br>(3.143-4.739)	1.514####<br>(1.372-1.912)	2.563****<br>(2.189-3.754)	1.68####<br>(1.405-2.021)	2.123**^^<br>(1.95-2.649)	1.493####<br>(1.197-1.86)	1.837^^^^<br>(1.625-1.934)	<0.0001
Cholesterol (mg/dL)	175<br>(170.3-174.9)	190<br>(179.5-197.5)	217***<br>(200-223.2)	175<br>(157.8-178.9)	193.5**<br>(180.9-204.3)	176<br>(161.1-182.9)	180.5<br>(169.6-192.5)	175<br>(164.2-186.1)	189.5<br>(182.2-204.7)	176<br>(160.4-181.5)	175^^^<br>(166.3-183.5)	<0.0001
TG (mg/dL)	135<br>(132.9-139.3)	127<br>(109-143.1)	149<br>(142.5-187.9)	103*<br>(96.25-129.5)	136<br>(124.1-175.6)	102***<br>(87.36-115.1)	136<br>(123.8-163.4)	90***<br>(81.24-108.7)	129<br>(118.5-142.9)	87.5****#<br>(75.24-104.5)	102^^^<br>(94.76-114.7)	<0.0001
LDL (mg/dL)	119<br>(118-125.7)	125<br>(118.8-133.8)	145**<br>(140.5-155.6)	111<br>(102.2-117.8)	119.5^^<br>(108.2-130.4)	109<br>(101.9-122.9)	113^^^<br>(102.5-126.1)	109<br>(102.1-125.6)	106^^^^<br>(101.9-121.4)	112<br>(99.61-114.3)	100**^^^^<br>(94.07-112.5)	<0.0001
HDL (mg/dL)	60<br>(59.08-75.86)	47.5***<br>(44.73-53.08)	46****<br>(42.59-51.76)	41****<br>(39.79-54.56)	45****<br>(41.32-49.55)	45****<br>(37.89-61.06)	47***<br>(44.35-52.38)	50**<br>(45.32-53.78)	52<br>(48.64-59.55)	52<br>(49.38-57.67)	55<br>(52.62-58.77)	<0.0001
Crea (mg/dL)	0.74<br>(0.727-0.783)	0.73<br>(0.708-0.757)	0.73<br>(0.684-0.754)	0.76<br>(0.723-0.801)	0.76<br>(0.737-0.845)	0.72<br>(0.714-0.797)	0.725<br>(0.711-0.796)	0.71<br>(0.699-0.772)	0.72<br>(0.698-0.762)	0.76<br>(0.726-0.821)	0.75<br>(0.721-0.797)	0.6237
UA (mg/dL)	3.925<br>(3.877-4.428)	6.01****<br>(5.435-6.912)	7.21****<br>(6.357-7.628)	5.215***<br>(4.947-6.203)	6.1****<br>(5.729-6.826)	4.69<br>(4.442-5.596)	5.58****<br>(5.156-6.062)	4.44<br>(4.162-4.956)	5.01*^<br>(4.789-5.41)	4.12####<br>(3.853-4.5)	4.595^^^^<br>(4.347-4.938)	<0.0001

**Urea (mg/dL)**	24.5<br>(23.73-26.95)	27<br>(25.83-30.89)	29<br>(27.16-34.2)	24<br>(19.81-25.49)	26<br>(24.87-30.87)	22<br>(19.52-26.38)	25.5<br>(23.47-28.8)	24<br>(22.33-29.34)	25<br>(23.26-29.18)	22.5<br>(21.64-28.03)	25<br>(23.14-30.36)	0.0032
**RBC (10^6/μL)**	4.7<br>(4.937-29.76)	4.73<br>(4.506-4.923)	4.51<br>(4.419-4.823)	4.76<br>(4.667-5.075)	4.59<br>(4.608-4.926)	4.655<br>(4.513-4.724)	4.755<br>(4.58-4.889)	4.63<br>(4.47-4.834)	4.77<br>(4.504-4.925)	4.62<br>(4.556-4.824)	4.485<br>(4.279-4.785)	0.5687
**HGB (g/dL)**	13.9<br>(13.94-37.39)	13<br>(12.62-13.64)	13.15<br>(12.99-14.06)	13.2<br>(12.69-13.63)	13.7<br>(13.23-14.26)	13.1<br>(12.88-13.53)	13.2<br>(12.64-13.86)	13.3<br>(12.75-13.75)	13.4<br>(12.89-14.03)	13.5<br>(12.84-13.93)	14.1<br>(13.04-14.66)	0.0193
**WBC (10^3/μL)**	7.5<br>(7.531-31.96)	7.87<br>(7.643-8.836)	**9.8***<br>(8.791-10.32)	6.45<br>(5.992-7.258)	7.16<br>(6.914-8.437)	**5.7**####**<br>(5.516-6.24)	7.43<br>(7.021-8.64)	5.95<br>(5.912-7.056)	7.91<br>(6.745-8.579)	**5.82**####**<br>(5.403-6.299)	**7.28^^**<br>(6.16-7.838)	<0.0001
**PLT (10^3/μL)**	287<br>(270.1-290.4)	262<br>(240.6-307.6)	**251****<br>(237.6-290.8)	254<br>(222.5-280.9)	226<br>(205.6-254.6)	265.5<br>(229.8-272.2)	250<br>(219.4-263.6)	284<br>(251-310.6)	256<br>(237.5-295)	**199****<br>(192.2-250.5)	**224***<br>(208.6-255.5)	0.0002
**CRP (mg/L)**	5.5<br>(5.438-5.667)	8.05<br>(6.635-13.92)	**11.55*****<br>(9.259-12.41)	5<br>(3.765-9.062)	7.8<br>(6.311-8.82)	6.1<br>(4.082-7.413)	6.9<br>(5.989-9.089)	5.1<br>(3.103-7.09)	**5.7^**<br>(4.762-6.919)	**4.9##**<br>(2.737-4.732)	**5.4^^**<br>(4.251-7.092)	<0.0001
**ALT (IU/L)**	24.5<br>(23.74-27.01)	25<br>(22.19-31.99)	27<br>(25.02-34.32)	25<br>(22.77-33.58)	28<br>(25.12-35.84)	20<br>(16.88-24.64)	24<br>(21.23-29.77)	**16**#**<br>(14.87-23.38)	21<br>(19.3-24.01)	**17.5**#**<br>(15-21)	19<br>(17.64-24.36)	<0.0001
**AST (IU/L)**	23<br>(22.71-25.83)	19.5<br>(18.22-26.18)	21<br>(20.54-29.65)	25<br>(19.7-35.64)	31<br>(25.19-39.85)	18<br>(16.46-22.28)	20<br>(18.87-25.89)	**17***<br>(15.13-22.57)	19.5<br>(17.75-25.3)	17<br>(17.17-25.31)	19<br>(17.9-23.85)	<0.0001

Table S2. Correlations between the analyzedoxidative/nitrosative stress and clinical parameters in patients with morbid obesity (OB) at the beginning of the study. Alanine transaminase (ALT). aspartate transaminase (AST). body mass index (BMI). C-reactive protein (CRP). creatinine (Crea). diastolic blood pressure (DBP). high-density lipoprotein (HDL). hemoglobin (HGB). homeostatic model assessment of insulin resistance (HOMA-IR). ischemia modified albumin (IMA). low-density lipoprotein (LDL). myeloperoxidase (MPO) nitric oxide (NO). red blood cell count (RBC). systolic blood pressure (SBP). triacylglycerol (TG). uric acid (UA). white blood cell count (WBC). waist-hip ratio (WHR).

	Dityrosine	Kynurenine	N-formyl-kynurenine	Tryptophan	Amyloid	Amadori Products	Glycophore	Total thiols	IMA	MPO	Total NO	Peroxynitrite	S-nitrosothiols	Nitrotyrosine
**BMI**	R=-0.252<br>p= 0.08	-0.33<br>p= 0.022	R=0.258<br>p= 0.073	R=-0.029<br>p= 0.845	R=-0.056<br>p= 0.709	R=0.324<br>p= 0.025	R=0.241<br>p= 0.092	R=-0.093<br>p= 0.529	R=0.08<br>p= 0.587	R=0.019<br>p= 0.898	R=0.002<br>p= 0.991	R=0.088<br>p= 0.557	R=0.066<br>p= 0.657	R=0.125<br>p= 0.387
**WHR**	R=-0.001<br>p= 0.996	R=-0.064<br>p= 0.666	R=-0.022<br>p= 0.879	R=0.053<br>p= 0.718	R=-0.1<br>p= 0.505	R=0.356<br>p= 0.013	R=-0.1<br>p= 0.49	R=0.249<br>p= 0.088	R=0.023<br>p= 0.877	R=-0.122<br>p= 0.409	R=0.076<br>p= 0.604	R=0.009<br>p= 0.952	R=0.074<br>p= 0.623	R=0.008<br>p= 0.954
**SBP**	R=-0.048<br>p= 0.744	R=-0.155<br>p= 0.292	R=0.042<br>p= 0.776	R=-0.022<br>p= 0.882	R=-0.033<br>p= 0.828	R=0.133<br>p= 0.368	R=-0.017<br>p= 0.904	R=0.024<br>p= 0.873	R=0.022<br>p= 0.881	R=-0.052<br>p= 0.724	R=-0.044<br>p= 0.766	R=0.164<br>p= 0.27	R=-0.227<br>p= 0.125	R=0.016<br>p= 0.91
**DBP**	R=0.205<br>p= 0.158	R=-0.051<br>p= 0.733	R=0.045<br>p= 0.761	R=-0.102<br>p= 0.484	R=-0.017<br>p= 0.908	R=0.066<br>p= 0.657	R=0.241<br>p= 0.092	R=-0.036<br>p= 0.808	R=0.067<br>p= 0.652	R=-0.067<br>p= 0.649	R=0.067<br>p= 0.648	R=0.198<br>p= 0.182	R=0.098<br>p= 0.514	R=0.005<br>p= 0.972
**Glucose**	R=0.195<br>p= 0.199	R=0.051<br>p= 0.742	R=0.224<br>p= 0.134	R=-0.107<br>p= 0.485	R=-0.088<br>p= 0.571	R=0.166<br>p= 0.276	R=0.104<br>p= 0.494	R=-0.185<br>p= 0.225	R=-0.172<br>p= 0.259	R=-0.009<br>p= 0.955	R=0.026<br>p= 0.864	R=0.247<br>p= 0.106	R=0.268<br>p= 0.078	R=-0.248<br>p= 0.097
**Insulin**	R=0.263<br>p= 0.081	R=0.147<br>p= 0.342	R=0.175<br>p= 0.244	R=-0.149<br>p= 0.329	R=0.035<br>p= 0.825	R=0.363<br>p= 0.016	R=0.005<br>p= 0.975	R=-0.299<br>p= 0.049	R=-0.127<br>p= 0.404	R=-0.172<br>p= 0.259	R=0.088<br>p= 0.566	R=0.326<br>p= 0.031	R=-0.16<br>p= 0.294	R=-0.163<br>p= 0.279
**HOMA-IR**	R=0.138<br>p= 0.385	R=0.102<br>p= 0.527	R=0.39<br>p= 0.01	R=-0.06<br>p= 0.706	R=-0.101<br>p= 0.534	R=0.37<br>p= 0.017	R=-0.004<br>p= 0.978	R=-0.306<br>p= 0.051	R=-0.055<br>p= 0.728	R=-0.144<br>p= 0.363	R=0.169<br>p= 0.285	R=0.101<br>p= 0.53	R=-0.083<br>p= 0.605	R=0.433<br>p= 0.004
**Cholesterol**	R=-0.111<br>p= 0.458	R=-0.161<br>p= 0.287	R=0.172<br>p= 0.248	R=-0.035<br>p= 0.816	R=-0.254<br>p= 0.093	R=0.05<br>p= 0.742	R=0.083<br>p= 0.575	R=-0.155<br>p= 0.304	R=0.137<br>p= 0.363	R=0.302<br>p= 0.041	R=-0.011<br>p= 0.942	R=0.138<br>p= 0.366	R=-0.067<br>p= 0.664	R=0.041<br>p= 0.784
**TG**	R=-0.256<br>p= 0.09	R=-0.271<br>p= 0.075	R=-0.046<br>p= 0.763	R=-0.044<br>p= 0.774	R=-0.119<br>p= 0.445	R=-0.027<br>p= 0.864	R=-0.078<br>p= 0.605	R=-0.042<br>p= 0.789	R=-0.019<br>p= 0.902	R=0.208<br>p= 0.175	R=0.33*<br>p= 0.027	R=0.298<br>p= 0.052	R=-0.101<br>p= 0.519	R=0.031<br>p= 0.837

**LDL**	R=-0.038<br>p= 0.805	R=-0.154<br>p= 0.318	R=0.136<br>p= 0.368	R=-0.031<br>p= 0.839	R=0.066<br>p= 0.675	R=0.17<br>p= 0.269	R=-0.092<br>p= 0.545	R=-0.267<br>p= 0.079	R=0.262<br>p= 0.082	R=0.163<br>p= 0.284	R=-0.055<br>p= 0.718	R=-0.002<br>p= 0.99	R=-0.246<br>p= 0.107	R=0.097<br>p= 0.52
**HDL**	R=0.083<br>p= 0.052	R=0.185<br>p= 0.235	R=0.035<br>p= 0.82	R=-0.103<br>p= 0.505	R=-0.114<br>p= 0.473	R=0.037<br>p= 0.813	R=-0.1<br>p= 0.515	R=-0.092<br>p= 0.557	R=0.282<br>p= 0.067	R=0.107<br>p= 0.495	R=-0.1<br>p= 0.517	R=-0.028<br>p= 0.862	R=0.081<br>p= 0.609	R=0.034<br>p= 0.822
**Crea**	R=-0.075<br>p= 0.593	R=-0.139<br>p= 0.344	R=0.004<br>p= 0.98	R=-0.065<br>p= 0.656	R=-0.17<br>p= 0.254	R=-0.283<br>p= 0.051	R=-0.075<br>p= 0.603	R=0.216<br>p= 0.141	R=-0.026<br>p= 0.861	R=0.072<br>p= 0.626	R=0.049<br>p= 0.739	R=-0.22<br>p= 0.138	R=-0.108<br>p= 0.47	R=0.104<br>p= 0.472
**UA**	R=-0.214<br>p= 0.607	R=-0.208<br>p= 0.157	R=0.187<br>p= 0.197	R=-0.053<br>p= 0.72	R=-0.223<br>p= 0.131	R=0.115<br>p= 0.437	R=-0.16<br>p= 0.268	R=-0.136<br>p= 0.357	R=-0.092<br>p= 0.535	R=-0.084<br>p= 0.57	R=0.179<br>p= 0.22	R=0.306<br>p= 0.036	R=0.056<br>p= 0.71	R=-0.011<br>p= 0.938
**Urea**	R=0.109<br>p= 0.454	R=0.181<br>p= 0.218	R=0.357<br>p= 0.012	R=-0.16<br>p= 0.273	R=-0.022<br>p= 0.883	R=-0.092<br>p= 0.534	R=-0.269<br>p= 0.059	R=-0.254<br>p= 0.081	R=-0.035<br>p= 0.814	R=0.049<br>p= 0.743	R=0.134<br>p= 0.357	R=0.088<br>p= 0.554	R=-0.2<br>p= 0.178	R=-0.135<br>p= 0.349
**RBC**	R=-0.285<br>p= 0.052	R=-0.127<br>p= 0.4	R=0.132<br>p= 0.376	R=0.089<br>p= 0.552	R=-0.18<br>p= 0.238	R=0.143<br>p= 0.343	R=-0.064<br>p= 0.664	R=0.063<br>p= 0.675	R=-0.179<br>p= 0.233	R=-0.254<br>p= 0.088	R=-0.132<br>p= 0.378	R=-0.057<br>p= 0.711	R=0.03<br>p= 0.844	R=-0.08<br>p= 0.587
**HGB**	R=-0.255<br>p= 0.091	R=-0.212<br>p= 0.167	R=0.095<br>p= 0.535	R=0.09<br>p= 0.55	R=-0.091<br>p= 0.564	R=0.078<br>p= 0.617	R=-0.064<br>p= 0.672	R=0.218<br>p= 0.156	R=-0.233<br>p= 0.128	R=-0.259<br>p= 0.09	R=-0.415<br>p= 0.005	R=-0.072<br>p= 0.644	R=0.102<br>p= 0.515	R=0.139<br>p= 0.356
**WBC**	R=-0.042<br>p= 0.775	R=-0.173<br>p= 0.241	R=0.098<br>p= 0.504	R=0.007<br>p= 0.96	R=-0.02<br>p= 0.895	R=0.049<br>p= 0.739	R=-0.131<br>p= 0.365	R=-0.354<br>p= 0.013	R=-0.061<br>p= 0.682	R=0.065<br>p= 0.662	R=-0.091<br>p= 0.536	R=-0.116<br>p= 0.436	R=0.012<br>p= 0.934	R=-0.021<br>p= 0.887
**CRP**	R=0.01<br>p= 0.948	R=0.132<br>p= 0.383	R=0.039<br>p= 0.792	R=-0.015<br>p= 0.918	R=-0.062<br>p= 0.686	R=0.212<br>p= 0.157	R=0.155<br>p= 0.292	R=-0.074<br>p= 0.626	R=-0.049<br>p= 0.741	R=0.012<br>p= 0.936	R=0.229<br>p= 0.122	R=0.311<br>p= 0.035	R=0.087<br>p= 0.565	R=0.074<br>p= 0.616
**ALT**	R=-0.038<br>p= 0.804	R=0.041<br>p= 0.79	R=0.001<br>p= 0.995	R=-0.133<br>p= 0.378	R=0.123<br>p= 0.425	R=-0.195<br>p= 0.199	R=-0.151<br>p= 0.31	R=-0.039<br>p= 0.802	R=-0.234<br>p= 0.118	R=-0.074<br>p= 0.626	R=-0.15<br>p= 0.319	R=-0.136<br>p= 0.375	R=-0.087<br>p= 0.57	R=-0.052<br>p= 0.726
**AST**	R=0<br>p= 0.998	R=0.03<br>p= 0.85	R=0.03<br>p= 0.855	R=0.16<br>p= 0.325	R=0.163<br>p= 0.315	R=-0.164<br>p= 0.311	R=0.017<br>p= 0.915	R=0.271<br>p= 0.091	R=-0.129<br>p= 0.428	R=0.042<br>p= 0.799	R=0.057<br>p= 0.728	R=-0.07<br>p= 0.672	R=0.091<br>p= 0.581	R=0<br>p= 1

Table S3. Correlations between the analyzed oxidative/nitrosative stress and clinical parameters in patients with morbid obesity (OB) at the end of the study. Alanine transaminase (ALT). aspartate transaminase (AST). body mass index (BMI). C-reactive protein (CRP). creatinine (Crea). diastolic blood pressure (DBP). high-density lipoprotein (HDL). hemoglobin (HGB). homeostatic model assessment of insulin resistance (HOMA-IR). ischemia modified albumin (IMA). low-density lipoprotein (LDL). myeloperoxidase (MPO) nitric oxide (NO). red blood cell count (RBC). systolic blood pressure (SBP). triacylglycerol (TG). uric acid (UA). white blood cell count (WBC). waist-hip ratio (WHR).

	**Dityrosine**	**Kynurenine**	**N-formyl-kynurenine**	**Tryptophan**	**Amyloid**	**Amadori Products**	**Glycophore**	**Total thiols**	**IMA**	**MPO**	**Total NO**	**Peroxynitrite**	**S-nitrosothiols**	**Nitrotyrosine**
**BMI**	R=-0.135<br>p= 0.376	R=-0.093<br>p= 0.555	R=0.082<br>p= 0.597	R=-0.201<br>p= 0.19	R=-0.033<br>p= 0.828	R=0.394<br>p= 0.007	R=0.182<br>p= 0.227	R=-0.018<br>p= 0.908	R=0.178<br>p= 0.249	R=0.386<br>p= 0.011	R=0.068<br>p= 0.654	R=-0.007<br>p= 0.962	R=0.11<br>p= 0.482	R=-0.018<br>p= 0.904
**Weight loss**	R= -0.334<br>p= 0.025	R= -0.175<br>p= 0.256	R= -0.293<br>p= 0.053	R= 0.288<br>p= 0.058	R= -0.075<br>p= 0.623	R= 0.104<br>p= 0.496	R= -0.017<br>p= 0.909	R= -0.024<br>p= 0.875	R= -0.123<br>p= 0.428	R= -0.174<br>p= 0.265	R= -0.233<br>p= 0.12	R= -0.028<br>p= 0.857	R= -0.133<br>p= 0.397	R= 0.11<br>p= 0.466
**WHR**	R=-0.162<br>p= 0.317	R=-0.121<br>p= 0.458	R=0.235<br>p= 0.149	R=-0.25<br>p= 0.125	R=-0.334<br>p= 0.033	R=0.168<br>p= 0.299	R=0.154<br>p= 0.335	R=0.02<br>p= 0.901	R=-0.18<br>p= 0.273	R=0.236<br>p= 0.154	R=0.0003484<br>p= 0.998	R=0.147<br>p= 0.379	R=-0.037<br>p= 0.824	R=-0.258<br>p= 0.104
**SBP**	R=0.005<br>p= 0.975	R=-0.091<br>p= 0.563	R=0.227<br>p= 0.134	R=0.042<br>p= 0.782	R=0.127<br>p= 0.408	R=0.146<br>p= 0.339	R=-0.057<br>p= 0.708	R=-0.165<br>p= 0.278	R=-0.159<br>p= 0.298	R=-0.078<br>p= 0.611	R=0.215<br>p= 0.152	R=-0.265<br>p= 0.079	R=-0.113<br>p= 0.458	R=0.185<br>p= 0.217
**DBP**	R=0.065	R=-0.339	R=0.303	R=-0.136	R=-0.112	R=0.141	R=-0.096	R=0.006	R=-0.136	R=-0.02	R=0.046	R=-0.153	R=-0.208	R=0.123

	p= 0.673	p= 0.026	p= 0.043	p= 0.373	p= 0.465	p= 0.355	p= 0.525	p= 0.969	p= 0.373	p= 0.895	p= 0.759	p= 0.315	p= 0.169	p= 0.414
**Glucose**	R=-0.171<br>p= 0.284	R=0.283<br>p= 0.081	R=0.109<br>p= 0.49	R=-0.044<br>p= 0.786	R=-0.001<br>p= 0.997	R=0.065<br>p= 0.688	R=0.03<br>p= 0.848	R=-0.355<br>p= 0.023	R=-0.123<br>p= 0.438	R=-0.182<br>p= 0.255	R=-0.059<br>p= 0.709	R=-0.065<br>p= 0.687	R=-0.057<br>p= 0.723	R=0.055<br>p= 0.731
**Insulin**	R=-0.073<br>p= 0.638	R=0.079<br>p= 0.618	R=-0.147<br>p= 0.348	R=0.072<br>p= 0.644	R=0.04<br>p= 0.799	R=-0.23<br>p= 0.133	R=0.231<br>p= 0.127	R=0.209<br>p= 0.174	R=-0.225<br>p= 0.147	R=-0.276<br>p= 0.077	R=0.106<br>p= 0.487	R=-0.329<br>p= 0.034	R=0.011<br>p= 0.945	R=0.143<br>p= 0.35
**HOMA-IR**	R=-0.121<br>p= 0.456	R=0.3<br>p= 0.067	R=-0.084<br>p= 0.6	R=0.149<br>p= 0.36	R=0.019<br>p= 0.909	R=-0.176<br>p= 0.278	R=0.193<br>p= 0.226	R=0.003<br>p= 0.983	R=-0.355<br>p= 0.023	R=-0.422<br>p= 0.007	R=0.013<br>p= 0.936	R=-0.339<br>p= 0.032	R=0.011<br>p= 0.946	R=0.174<br>p= 0.277
**Cholesterol**	R=0.015<br>p= 0.924	R=0.2<br>p= 0.193	R=-0.239<br>p= 0.113	R=0.139<br>p= 0.361	R=0.073<br>p= 0.631	R=-0.075<br>p= 0.618	R=0.242<br>p= 0.101	R=0.091<br>p= 0.549	R=-0.05<br>p= 0.744	R=0.001<br>p= 0.997	R=0.155<br>p= 0.299	R=-0.166<br>p= 0.28	R=0.167<br>p= 0.279	R=0.082<br>p= 0.584
**TG**	R=-0.007<br>p= 0.963	R=0.21<br>p= 0.172	R=-0.082<br>p= 0.593	R=-0.127<br>p= 0.406	R=0.264<br>p= 0.076	R=-0.007<br>p= 0.964	R=0.085<br>p= 0.568	R=-0.101<br>p= 0.502	R=-0.029<br>p= 0.85	R=0.102<br>p= 0.51	R=0.082<br>p= 0.585	R=-0.02<br>p= 0.899	R=-0.107<br>p= 0.49	R=0.118<br>p= 0.431
**LDL**	R=-0.244<br>p= 0.103	R=0.294<br>p= 0.053	R=-0.374<br>p= 0.011	R=0.281<br>p= 0.061	R=0.117<br>p= 0.438	R=-0.082<br>p= 0.587	R=0.112<br>p= 0.453	R=0.087<br>p= 0.567	R=0.16<br>p= 0.293	R=-0.055<br>p= 0.724	R=-0.076<br>p= 0.61	R=-0.081<br>p= 0.601	R=0.289<br>p= 0.057	R=0.171<br>p= 0.251
**HDL**	R=0.236<br>p= 0.118	R=-0.029<br>p= 0.856	R=-0.002<br>p= 0.992	R=0.27<br>p= 0.076	R=0.079<br>p= 0.605	R=-0.086<br>p= 0.574	R=-0.266<br>p= 0.074	R=0.072<br>p= 0.638	R=-0.152<br>p= 0.325	R=-0.234<br>p= 0.132	R=0.069<br>p= 0.649	R=0.001<br>p= 0.995	R=0.042<br>p= 0.79	R=-0.129<br>p= 0.393
**Crea**	R=0.168<br>p= 0.258	R=-0.073<br>p= 0.633	R=0.133<br>p= 0.374	R=-0.19<br>p= 0.205	R=0.042<br>p= 0.779	R=-0.13<br>p= 0.384	R=0.193<br>p= 0.189	R=-0.16<br>p= 0.282	R=0.272<br>p= 0.064	R=0.053<br>p= 0.725	R=0.112<br>p= 0.45	R=0.248<br>p= 0.096	R=-0.32<br>p= 0.03	R=-0.111<br>p= 0.454
**UA**	R=0.139<br>p= 0.355	R=-0.069<br>p= 0.655	R=0.149<br>p= 0.322	R=-0.171<br>p= 0.26	R=-0.063<br>p= 0.68	R=0.155<br>p= 0.302	R=-0.034<br>p= 0.82	R=0.169<br>p= 0.261	R=-0.015<br>p= 0.921	R=-0.018<br>p= 0.908	R=-0.163<br>p= 0.274	R=0.016<br>p= 0.919	R=0.303<br>p= 0.043	R=0.143<br>p= 0.339
**Urea**	R=-0.131<br>p= 0.38	R=-0.133<br>p= 0.379	R=0.172<br>p= 0.252	R=-0.101<br>p= 0.505	R=-0.17<br>p= 0.253	R=0.268<br>p= 0.069	R=-0.031<br>p= 0.834	R=-0.322<br>p= 0.027	R=-0.137<br>p= 0.363	R=0.021<br>p= 0.89	R=-0.115<br>p= 0.437	R=0.073<br>p= 0.634	R=0.157<br>p= 0.302	R=0.14<br>p= 0.342
**RBC**	R=-0.251<br>p= 0.1	R=0.447<br>p= 0.003	R=-0.175<br>p= 0.261	R=0.364<br>p= 0.016	R=0.101<br>p= 0.515	R=-0.111<br>p= 0.472	R=0.145<br>p= 0.343	R=-0.311<br>p= 0.04	R=0.038<br>p= 0.808	R=-0.162<br>p= 0.304	R=0.005<br>p= 0.975	R=0.097<br>p= 0.541	R=0.241<br>p= 0.123	R=0.104<br>p= 0.497
**HGB**	R=-0.064<br>p= 0.679	R=0.015<br>p= 0.922	R=-0.07<br>p= 0.655	R=-0.083<br>p= 0.598	R=-0.143<br>p= 0.355	R=0.161<br>p= 0.295	R=0.165<br>p= 0.28	R=0.05<br>p= 0.748	R=0.032<br>p= 0.838	R=0.191<br>p= 0.226	R=-0.007<br>p= 0.965	R=-0.023<br>p= 0.886	R=0.263<br>p= 0.092	R=0.074<br>p= 0.628
**WBC**	R=-0.008<br>p= 0.958	R=0.056<br>p= 0.723	R=-0.016<br>p= 0.921	R=0.183<br>p= 0.239	R=-0.004<br>p= 0.979	R=0.015<br>p= 0.923	R=0.164<br>p= 0.281	R=-0.143<br>p= 0.355	R=-0.233<br>p= 0.132	R=-0.114<br>p= 0.471	R=0.28<br>p= 0.063	R=-0.075<br>p= 0.636	R=0.213<br>p= 0.176	R=0.122<br>p= 0.424
**CRP**	R=0.066<br>p= 0.672	R=-0.134<br>p= 0.405	R=0.263<br>p= 0.093	R=-0.134<br>p= 0.393	R=0.074<br>p= 0.639	R=-0.027<br>p= 0.862	R=0.057<br>p= 0.712	R=-0.286<br>p= 0.063	R=-0.136<br>p= 0.392	R=-0.114<br>p= 0.477	R=0.046<br>p= 0.766	R=-0.073<br>p= 0.65	R=-0.209<br>p= 0.19	R=0.11<br>p= 0.476
**ALT**	R=0.071<br>p= 0.643	R=0.045<br>p= 0.775	R=-0.017<br>p= 0.912	R=0.121<br>p= 0.432	R=0.217<br>p= 0.153	R=-0.051<br>p= 0.74	R=0.132<br>p= 0.382	R=0.132<br>p= 0.387	R=-0.203<br>p= 0.187	R=-0.02<br>p= 0.9	R=-0.179<br>p= 0.234	R=-0.126<br>p= 0.42	R=-0.183<br>p= 0.241	R=0.087<br>p= 0.563
**AST**	R=-0.014<br>p= 0.93	R=0.327<br>p= 0.039	R=0.035<br>p= 0.823	R=0.01<br>p= 0.952	R=0.026<br>p= 0.872	R=-0.007<br>p= 0.965	R=0.127<br>p= 0.418	R=0.005<br>p= 0.976	R=-0.011<br>p= 0.945	R=-0.169<br>p= 0.289	R=-0.172<br>p= 0.27	R=-0.302<br>p= 0.055	R=0.087<br>p= 0.588	R=0.143<br>p= 0.359
